# FMF: an update

**DOI:** 10.1186/1546-0096-12-S1-I33

**Published:** 2014-09-17

**Authors:** Seza Ozen

**Affiliations:** 1 Pediatric Rheumatology, Hacettepe University, Ankara, Turkey

## 

Familial Mediterranean fever (FMF) is the most common autoinflammatory disease over the world. This autosomal recessively inherited disease is due to mutations in the gene coding for pyrin. Disease causing mutations in the gene are associated with excessive levels of IL-1. The clinical symptoms of inflammation are mainly in the form of fever and serositis along with laboratory evidence of persistently raised acute phase reactants, including serum amyloid A levels. Untreated patients suffer the consequences of chronic inflammation.

Patients who display inflammatory symptoms but who carry one mutation only should be carefully evaluated for the need of therapy. Colchicine is the main treatment of FMF. Management of the patients includes following clinical activity of the disease and acute phase reactants on a regular basis and checking for drug compliance, and monitoring side effects of the drug. If patients are intolerant to or unresponsive to colchicine anti IL1 treatment should be considered.

## Disclosure of interest

None declared.

